# PVP/ZIF-8-Derived Zn, Ni Co-loaded N-Doped Porous Carbon as a Catalyst for an Efficient Hydrogen Evolution Reaction

**DOI:** 10.3389/fchem.2020.00723

**Published:** 2020-10-14

**Authors:** Yanqiu Jing, Qiang Lei, Gang Hu, Jixian He, Xiao Lei, Fei Wang, Junju Li, Yide Yang, Xuewei Zhang

**Affiliations:** ^1^College of Tobacco Science, Henan Agricultural University, Zhengzhou, China; ^2^Sichuan of China National Tobacco Corporation, Chengdu, China; ^3^Raw Materials Supply Center of China Tobacco Guangdong Industrial Co., Ltd., Guangzhou, China

**Keywords:** ZIF-8, ZnNi/NPC, porous structure, Ni doping, hydrogen evolution reaction

## Abstract

Exploring catalysts with low cost and excellent performance for the hydrogen evolution reaction (HER) is still a significant challenge. In this work, zeolitic imidazolate framework 8 (ZIF-8), hybridized with polyvinylpyrrolidone, was used to prepare Zn, Ni co-loaded N-doped porous carbon (ZnNi/NPC) via a straightforward absorption and pyrolysis process. The as-prepared ZnNi/NPC was used as a catalyst for the HER. This experiment showed that the porous structure and Ni doping have a significant influence on the HER activity of the catalyst. Compared with Zn/NC and Zn/NPC, ZnNi/NPC exhibits superior HER activity with an overpotential of 198 mV and a Tafel slope of 69.2 mV dec^−1^. ZnNi/NPC also shows excellent physical and chemical stability during the HER process. Considering the lower cost and excellent HER performance of ZnNi/NPC, this work provides an attractive solution to fabricate non-precious materials and offers a possible new strategy to replace Pt-based electrocatalysts for HER.

## Introduction

Hydrogen is viewed as one of the most promising forms of energy to replace traditional fossil fuels because of its sustainable performance and high energy density (Chen N. et al., 2020). Water splitting is an efficient and straightforward route to obtaining hydrogen through the hydrogen evolution reaction (HER) (Qamar et al., 2016). However, many electrocatalysts have a high overpotential and large Tafel slope, which makes them exhibit sluggish HER kinetics and prevents their widespread use (Tian et al., 2017). Thus, there is an urgent need to explore electrocatalysts with excellent HER performance.

Currently, Pt-based materials are still considered to be the most effective electrocatalysts for HER because of their outstanding catalytic activity (Lao et al., 2019). However, these Pt-based materials are expensive and are in short supply, which impedes their widespread use. Many researchers have devoted themselves to exploring high-efficiency electrocatalysts, for example carbon-based materials, and have found that doping with some Earth-abundant non-precious transition metals, such as Mn, Fe, Ni, and Co, can improve the efficiency of the carbon-based materials. For example, Chen T. T. et al. (2020) used a Co-based metal–organic framework (MOF) containing nitrogen and oxygen heteroatoms (Co-NOMOF) mixed with thiomolybdate [Mo_3_S_13_]^2−^ nanoclusters as a precursor to preparing a Co_9_S_8_/MoS_2_@NSOC nanocomposite via a direct pyrolysis process. They found that Co_9_S_8_/MoS_2_@NSOC exhibits excellent HER performance with an overpotential of 194 and 233 mV in 1 M KOH and 0.5 M H_2_SO_4_ solution at 10 mA cm^−2^, respectively (Chen W. et al., 2019). Chu et al. (2018) reported a simple method of fabricating carbon-coated nickel–nickel oxide composites (Ni/NiO@C/GR-t-w) and found that Ni/NiO@C/GR-900-8 showed superior electrocatalytic properties with a lower overpotential of 108 mV (vs. HER) at 10 mA cm^−2^ and a smaller Tafel slope of 44 mV dec^−1^.

Zeolitic imidazolate framework 8 (ZIF-8), constructed using organic ligands and Zn^2+^, has a high specific surface area, adjustable pore size, and excellent physical and chemical stability. It is widely used for sensing (Chen W. et al., 2019; Knedel et al., 2020), drug delivery (Adhikari et al., 2015; Qin et al., 2019), and catalysis (Liu et al., 2014; Yang et al., 2018). It can also be directly pyrolyzed into carbon at high temperature under an N_2_/Ar atmosphere. In this paper, we used ZIF-8 as a precursor and polyvinylpyrrolidone (PVP) as a hard template to prepare Zn, Ni co-loaded N-doped porous carbon (ZnNi/NPC) via a straightforward absorption and pyrolysis process. We explored the influence of the porous structure and Ni doping on the electrocatalytic performance in HER and found that the porous structure and Ni doping can significantly improve the HER activity of Zn-loaded N-doped carbon. The excellent HER performance of ZnNi/NPC makes it suitable for practical applications. Furthermore, we hope this work can provide a theorical and practical basis for subsequent research.

## Experimental Section

### Materials

Zinc nitrate hexahydrate [Zn(NO_3_)_2_·6H_2_O], 2-methylimidazole [C_4_H_6_N_2_], nickel nitrate hexahydrate [Ni(NO_3_)_2_·6H_2_O], PVP (K30), ethanol, and methanol were of analytical grade and were purchased from Sinopharm Chemical Reagent Co., Ltd. Nafion solution (5%) was obtained from Sigma-Aldrich Chemical Co., Ltd. All reagents were used as received from the manufacturer without any further treatment.

### Fabrication of the ZnNi/NPC Electrocatalyst

**Figure 1:**
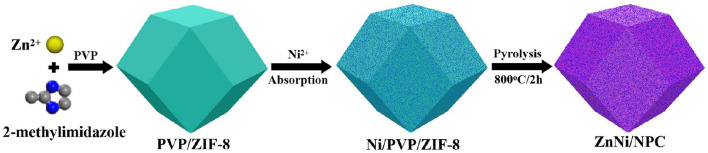
Schematic diagram for the fabrication of ZnNi/NPC.

In this experiment, ZnNi/NPC was fabricated by using the PVP/ZIF-8 composite as a precursor via a straightforward absorption and pyrolysis process. In a typical procedure, 3.3 g of Zn(NO_3_)_2_·6H_2_O was dissolved in 100 mL of methanol to form a homogeneous solution (solution A), and 3.0 g of 2-methylimidazole and 7 g of PVP were dissolved in a further 100 mL of methanol to form another homogeneous solution (solution B). Solution A was slowly added into solution B under vigorous stirring for 5 min. Next, the mixture was aged at room temperature for 20 h and a layer of white precipitate was formed. The white precipitate was centrifuged, washed with ethanol, and dried at 60°C for 6 h to obtain the PVP/ZIF-8 composite. Then, 1.0 g of PVP/ZIF-8 composite was dispersed in 30 mL of ethanol solution containing 0.2 g of Ni(NO_3_)_2_·6H_2_O under vigorous stirring for 30 min. The suspension was centrifuged and dried at 80°C for 10 h to obtain the Ni-loaded PVP/ZIF-8 composite. Finally, the Ni-loaded PVP/ZIF-8 composite was pyrolyzed at 800°C for 2 h at a heating rate of 2°C min^−1^ under an H_2_/Ar atmosphere [*V*(H_2_):*V*(Ar) = 1:9] to fabricate ZnNi/NPC. A schematic diagram for the fabrication of ZnNi/NPC is shown in Figure 1.

### Characterization

The X-ray diffraction (XRD) patterns of all as-synthesized samples were recorded on a Bruker D8 diffractometer with Cu Kα radiation (λ = 0.1541 nm) at 40 kV and 40 mA. Raman spectra were obtained with a Horiba Evolution spectrometer with a 532 nm excitation wavelength. Scanning electron microscopy (SEM) was carried out on a Hitachi SU70 scanning electron microscope. Transmission electron microscopy (TEM) and high-resolution TEM were carried out on a Tecnai F20 machine with an acceleration voltage of 200 kV. X-ray photoelectron spectra (XPS) were obtained by means of an Escalab 250Xi system (Thermo Scientific).

### Electrochemical Measurements

The HER performance of all as-synthesized samples was measured on a CHI 660E using a typical three-electrode setup. An Ag/AgCl electrode, Pt wire, and 1.0 M KOH solution were used as the reference electrode, the counter electrode, and the electrolyte, respectively. A glassy carbon electrode (GCE; 5 mm in diameter), supporting with catalyst, was used as the working electrode. Before preparing the working electrode was supported with catalyst, the GCE was sonicated with deionized water and dried at room temperature. Then, 25 μL of catalyst ink, consisting of 10 mg of catalyst, 180 μL of alcohol solution, and 5 μL of Nafion solution (5%), was dropped onto the surface of the GCE three times and fully dried at room temperature. Before the HER performance tests, the electrolyte was pre-purged and saturated with N_2_ to eliminate the influence of dissolved oxygen. The linear sweep voltammetry (LSV) measurements were performed from 0 to −0.6 V at a rotation rate of 1,600 rpm and scan rate of 2 mV s^−1^. The chronoamperometric curve of ZnNi/NPC was obtained at a voltage of 150 mV in N_2_-saturated 1.0 M KOH for 12 h.

## Results and Discussion

### XRD and Raman Spectra Analysis

**Figure 2:**
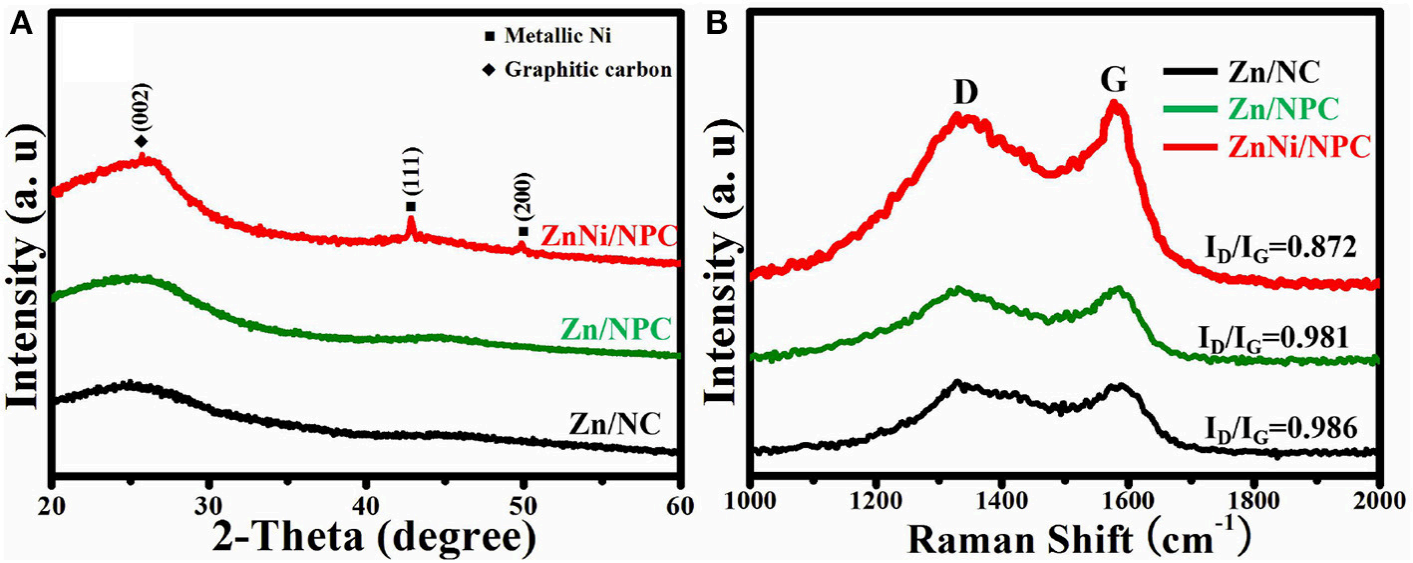
**(A)** XRD patterns and **(B)** Raman spectra of Zn/NC, Zn/NPC, and ZnNi/NPC.

The crystalline structure of all as-synthesized samples was characterized by XRD. As shown in Figure 2A, all samples exhibit a broad band at about 26°, which is ascribed to the characteristic peak of carbon (Zhou et al., 2016). The organic ligands are removed and transformed into carbon under high temperature. In addition to the characteristic peak of carbon, ZnNi/NPC also shows new peaks at 44.1° and 51.8°, which correspond to the (111) and (200) reflections of metallic Ni, respectively (Chu et al., 2018). Figure 2B shows the Raman spectra of Zn/NC, Zn/NPC, and ZnNi/NPC. All samples have two broad bands at about 1,328 cm^−1^ (G band) and 1,569 cm^−1^ (D band). According to previous reports, we know that the G band and D band are ascribed to the characteristic bands of amorphous carbon and graphitic carbon, respectively (Qing et al., 2010; Wang et al., 2014). The *I*_D_/*I*_G_ values of Zn/NC and Zn/NPC are almost the same. PVP cannot enhance the degree of graphitization of the catalyst. However, when the catalyst is loaded with Ni element, the *I*_D_/*I*_G_ value of the catalyst decreases to 0.872. Ni element can significantly improve the degree of graphitization of the catalyst, which is in accordance with previous reports (Xiang et al., 2014). As we know, the electrical conductivity of graphitic carbon is superior to that of amorphous carbon. This indicates that ZnNi/NPC can exhibit elevated HER activity.

### SEM Images

**Figure 3:**
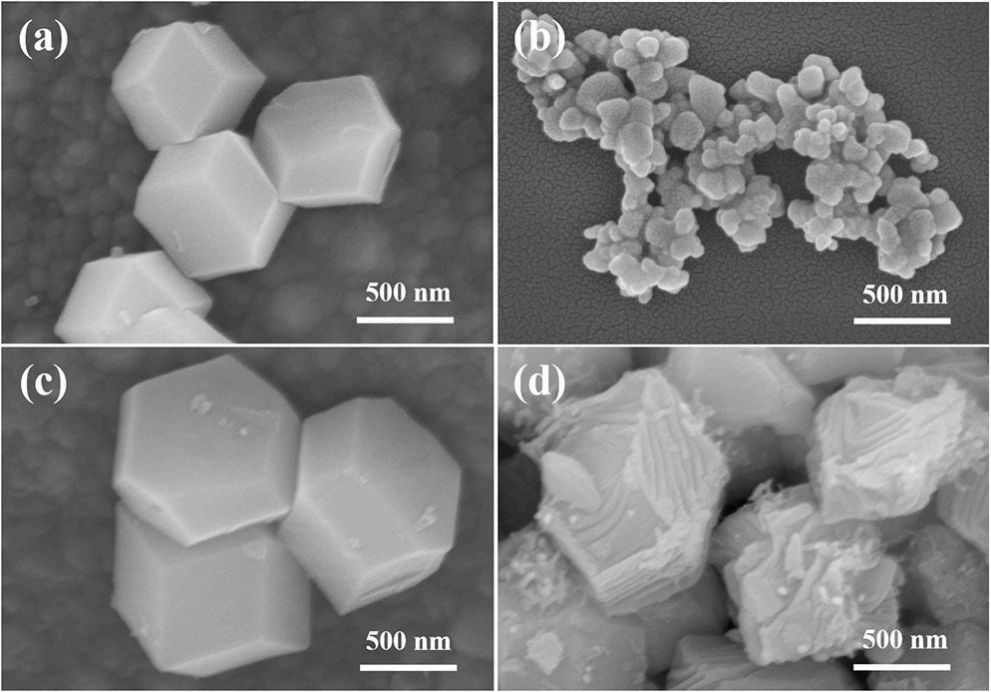
SEM images of **(a)** ZIF-8, **(b)** Zn/NC, **(c)** Zn/NPC, and **(d)** ZnNi/NPC.

Figure 3 shows SEM images of ZIF-8, Zn/NC, Zn/NPC, and ZnNi/NPC. As shown in Figure 3a, ZIF-8 exhibits a dodecahedral morphology. However, when ZIF-8 is treated at high temperature, its structure is broken down (Figure 3b). Figure 3c shows an SEM image of Zn/NPC nanoparticles, which also exhibit a dodecahedral morphology. As described in previous reports, PVP acts as a hard template, meaning that the ZIF-derived catalyst retains its original structure (Liu et al., 2016). Figure 3d shows an SEM image of ZnNi/NPC. ZnNi/NPC nanoparticles exhibit dodecahedral morphology. However, the surface of the ZnNi/NPC nanoparticles is etched. According to a report by Lou and co-workers, a zeolitic imidazolate framework can be corroded by Ni^2+^ during the hydrolysis process (He et al., 2017). This implies that Ni^2+^ can also corrode the structure of ZIF-8 during the hydrolysis process.

### TEM Images

**Figure 4:**
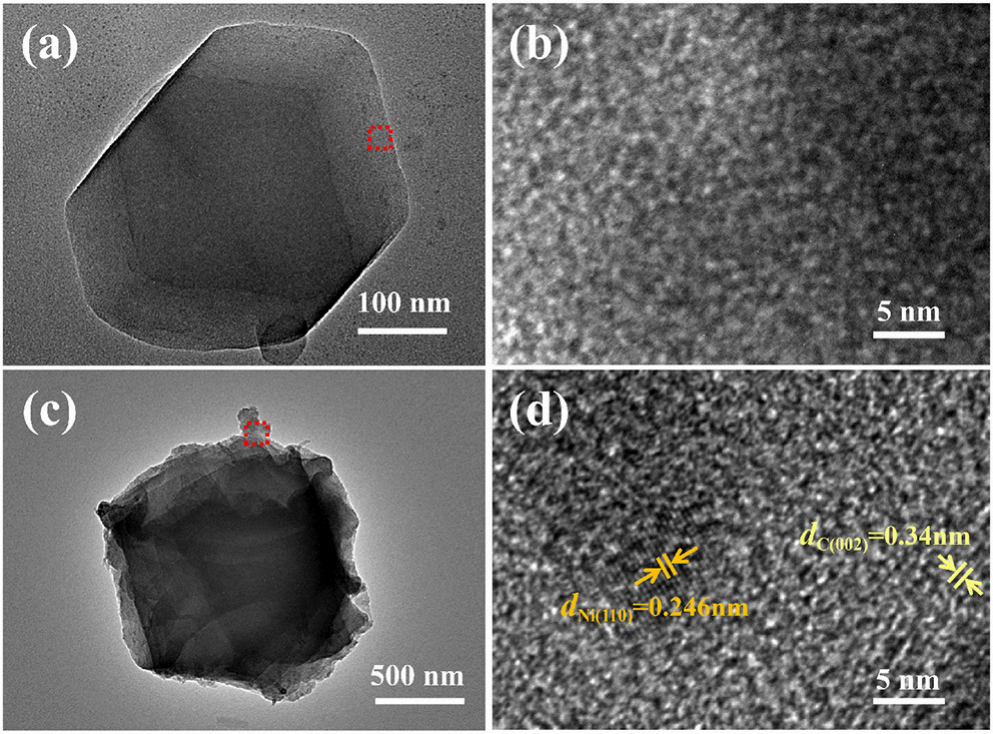
TEM images of **(a,b)** Zn/NPC and **(c,d)** ZnNi/NPC.

In order to further analyze the microstructure of the catalyst, TEM images were obtained by means of a Tecnai F20. As shown in Figure 4a, Zn/NPC nanoparticles exhibit a dodecahedral morphology, which is in agreement with the SEM image (Figure 3a). Figure 4b shows a higher magnification of Zn/NPC, demonstrating the porous structure. Figures 4c,d are the low-magnification and high-magnification images of ZnNi/NPC, respectively. The surface of the ZnNi/NPC is covered with wrinkles. In the internal structure, there are some nanoparticles that exhibit two different lattice fringes of 0.246 and 0.34 nm, which correspond to the (110) lattice plane of metallic Ni and the (002) lattice plane of graphitic carbon, respectively (Liu et al., 2017).

### XPS Analysis

**Figure 5:**
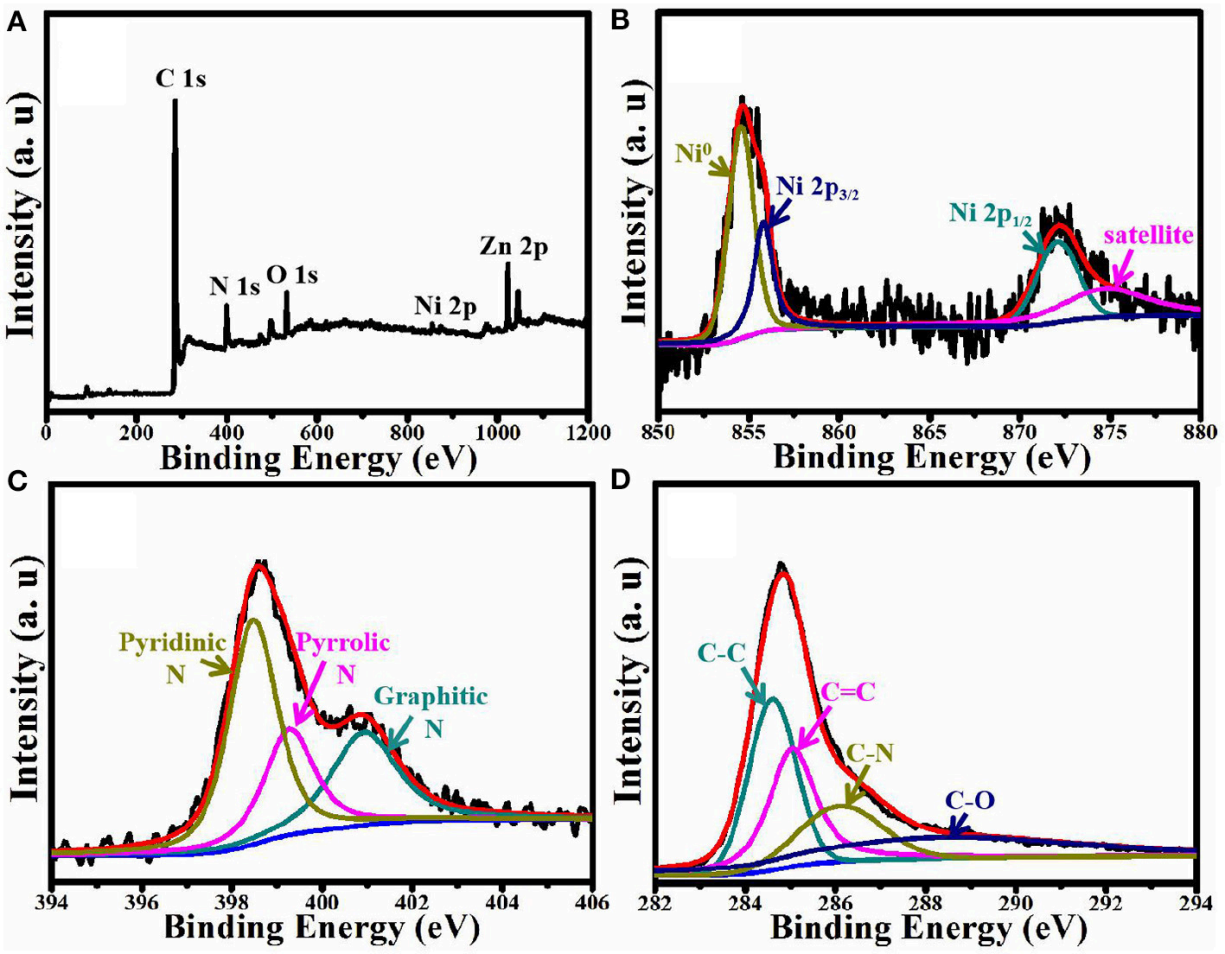
XPS spectra of ZnNi/NPC: **(A)** full XPS spectrum, **(B)** Ni 2p, **(C)** N 1s, and **(D)** C 1s.

The composition and chemical states of ZnNi/NPC were verified by XPS technology. As shown in Figure 5A, ZnNi/NPC is composed of elemental Zn, Ni, C, N, and O. Elemental O could come from the slight oxidation of the catalyst and/or the absorption of oxygen. Figure 5B shows the Ni 2p spectrum, which is deconvoluted into four peaks, corresponding to metallic Ni (Ni^0^), Ni 2p_3/2_, Ni 2p_1/2_, and a satellite. Figure 5C shows the N signal of ZnNi/NPC. The N 1s spectrum is deconvoluted into three peaks at 398.1, 399.3, and 401.7 eV, which are ascribed to pyridinic N, pyrrolic N, and graphitic N, respectively (Pan et al., 2018). Figure 5D shows the C 1s spectrum. The C 1s spectrum shows peaks at 284.3 and 285.1 eV, which correspond to the C–C band and C=C band, respectively. Furthermore, the C 1s spectrum also shows peaks at 286.2 and 288.7 eV, corresponding to the C–N band and C–O band, respectively. This confirms that the porous carbon base is doped with N and the catalyst is slightly oxidized.

### HER Performance

**Figure 6:**
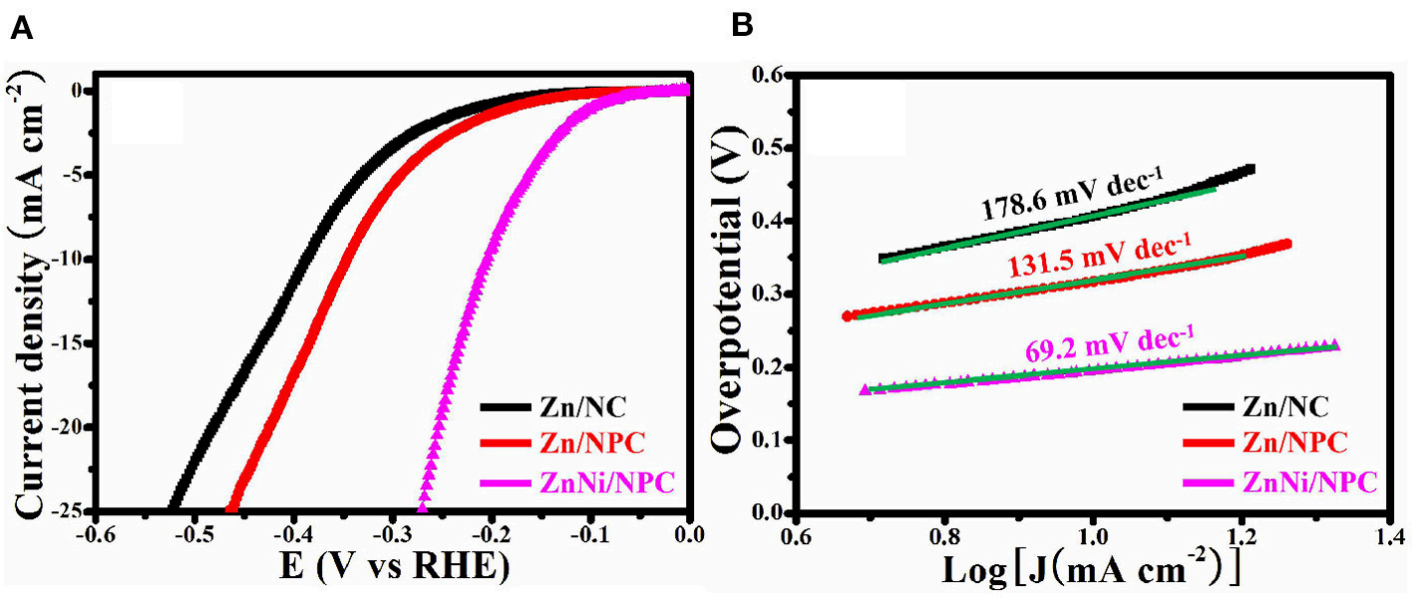
**(A)** LSV curves and **(B)** corresponding Tafel plots of Zn/NC, Zn/NPC, and ZnNi/NPC.

The HER performance of all as-synthesized samples was assessed by means of a typical three-electrode setup. Figure 6A shows the LSV curves of Zn/NC, Zn/NPC, and ZnNi/NPC. It can be seen that the structure and Ni doping have a great influence on the HER activity of the catalyst. Zn/NC exhibits inferior HER activity with an overpotential of 386 mV at 10 mA cm^−2^. However, Zn/NPC shows superior HER activity with an overpotential of 344 mV at 10 mA cm^−2^. The porous structure can improve the HER activity. Compared with Zn/NC and Zn/NPC, ZnNi/NPC displays the optimal HER activity with an overpotential of 198 mV at 10 mA cm^−2^. Figure 6B shows the Tafel plots corresponding to the LSV curves. Zn/NC, Zn/NPC, and ZnNi/NPC exhibit Tafel slopes of 178.6, 131.5, and 69.2 mV dec^−1^, respectively. ZnNi/NPC shows the lowest Tafel slope of 69.2 mV dec^−1^. This confirms that the porous structure and Ni doping can significantly improve the HER performance of the catalyst.

### Stability

**Figure 7:**
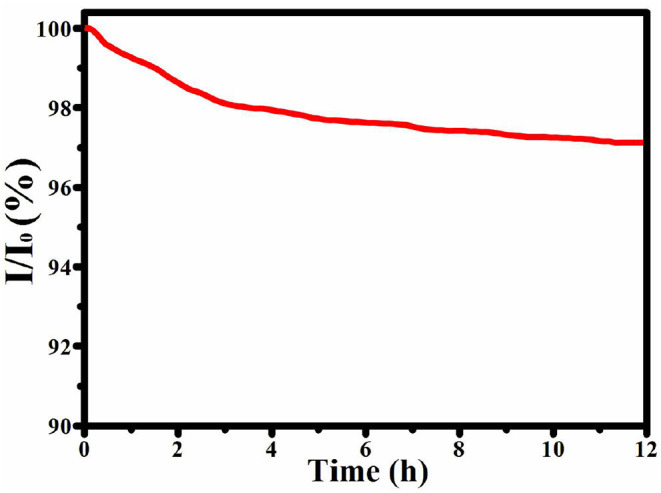
Chronoamperometric response of ZnNi/NPC at 150 mV.

The durability of the catalyst is also very important when it is used for practical applications. Therefore, ZnNi/NPC was measured at 150 mV in N_2_-saturated 1.0 M KOH at a rotation rate of 1,600 rpm for 12 h. As shown in Figure 7, the current density of ZnNi/NPC decreases by just 3.8%. This confirms that ZnNi/NPC has excellent physical and chemical stability during the HER process; this may be attributed to the tight attachment of Zn, Ni on porous carbon.

## Conclusion

In summary, metallic Ni and Zn were successfully co-loaded on N-doped porous carbon (ZnNi/NPC) by adopting PVP/ZIF-8 as the precursor via a straightforward absorption and pyrolysis process. The as-synthesized ZnNi/NPC was used as a catalyst for HER. Zn-loaded N-doped carbon (Zn/NC) exhibits inferior HER activity with an overpotential of 386 mV and a Tafel slope of 178.6 mV dec^−1^. However, Zn-loaded N-doped porous carbon (Zn/NPC) shows superior HER activity with an overpotential of 344 mV and a Tafel slope of 131.5 mV dec^−1^. Furthermore, Zn, Ni co-loaded N-doped porous carbon (ZnNi/NPC) displays the optimal HER activity with an overpotential of 198 mV and a Tafel slope of 69.2 mV dec^−1^. The porous structure and Ni doping can significantly improve the HER activity of Zn-loaded N-doped carbon. Furthermore, ZnNi/NPC exhibits robust stability for HER. After 12 h of continuous measurement, the current density of ZnNi/NPC decreased by just 3.8%. The excellent HER performance of ZnNi/NPC makes it a possibility for practical applications.

## Data Availability Statement

The raw data supporting the conclusions of this article will be made available by the authors, without undue reservation.

## Author Contributions

YJ, YY, and XZ designed the experiments and wrote the manuscript. QL, GH, JH, FW, JL, YY, and XL carried out the experiments. All authors contributed to the article and approved the submitted version.

## Conflict of Interest

QL, GH, JH, FW, JL, YY, and XL were employed by Sichuan of China National Tobacco Corporation. XZ was employed by Raw Materials Supply Centre of China Tobacco Guangdong Industrial Co., Ltd. The remaining author declares that the research was conducted in the absence of any commercial or financial relationships that could be construed as a potential conflict of interest.
